# On sufficient density conditions for lattice orbits of relative discrete series

**DOI:** 10.1007/s00013-022-01748-8

**Published:** 2022-05-23

**Authors:** Ulrik Enstad, Jordy Timo van Velthoven

**Affiliations:** 1 Department of Mathematics, Stockholm University, Stockholm, 106 91 Sweden; 2 Delft University of Technology, Mekelweg 4, Building 36, Delft, CD 2628 The Netherlands

**Keywords:** Density condition, Discrete series, Frame, Lattice, Riesz sequence, 22D25, 22E27, 42C30, 42C40

## Abstract

This note provides new criteria on a unimodular group *G* and a discrete series representation $(\pi , \mathcal {H}_{\pi })$ of formal degree $d_{\pi } > 0$ under which any lattice $\Gamma \le G$ with ${{\,\mathrm{vol}\,}}(G/\Gamma ) d_{\pi } \le 1$ (resp. ${{\,\mathrm{vol}\,}}(G/\Gamma ) d_{\pi } \ge 1$) admits $g \in \mathcal {H}_{\pi }$ such that $\pi (\Gamma ) g$ is a frame (resp. Riesz sequence). The results apply to all projective discrete series of exponential Lie groups.

## Introduction

Let *G* be a second-countable unimodular group with a lattice $\Gamma \le G$. For an irreducible projective unitary representation $(\pi , \mathcal {H}_{\pi })$ of *G*, let $\pi (\Gamma ) g$ be the $\Gamma$-orbit of $g \in \mathcal {H}_{\pi }$, i.e.,

$$\begin{aligned} \pi (\Gamma ) g = \big \{ \pi (\gamma ) g : \gamma \in \Gamma \big \}. \end{aligned}$$

An orbit $\pi (\Gamma ) g$ is said to be a *frame* for $\mathcal {H}_{\pi }$ if there exist constants $A, B >0$ such that

1.1 $$\begin{aligned} A \Vert f \Vert _{\mathcal {H}_{\pi }}^2 \le \sum _{\gamma \in \Gamma } | \langle f, \pi (\gamma ) g \rangle |^2 \le B \Vert f \Vert _{\mathcal {H}_{\pi }}^2, \quad f \in \mathcal {H}_{\pi }. \end{aligned}$$

A *Bessel sequence* is a system $\pi (\Gamma ) g$ satisfying the upper bound in (1.1). The lower bound in (1.1) implies, in particular, that *g* is a cyclic vector for the restriction $\pi |_{\Gamma }$.

A system $\pi (\Gamma ) g$ is a *Riesz sequence* if it satisfies inequalities dual to (1.1), namely

1.2 $$\begin{aligned} A \Vert c \Vert _{\ell ^2}^2 \le \bigg \Vert \sum _{\gamma \in \Gamma } c_{\gamma } \pi (\gamma ) g \bigg \Vert _{\mathcal {H}_{\pi }}^2 \le B \Vert c \Vert _{\ell ^2}^2, \quad c \in \ell ^2 (\Gamma ). \end{aligned}$$

If $\pi (\Gamma ) g$ satisfies the upper bound in (1.2), then it is a Bessel sequence. The lower bound in (1.2) implies, in particular, that a Riesz sequence is linearly independent.

This note is concerned with the existence of vectors $g \in \mathcal {H}_{\pi }$ such that its orbit $\pi (\Gamma ) g$ is a frame or Riesz sequence. A simple necessary condition is that if $\pi (\Gamma ) g$ is a frame or Riesz sequence, so that it admits a Bessel constant $B > 0$, then $g \in \mathcal {H}_{\pi }\setminus \{0\}$ satisfies

$$\begin{aligned} \int \limits _{G} | \langle g, \pi (x) g \rangle |^2 \; d\mu _G (x)= & {} \int \limits _{G/\Gamma } \sum _{\gamma \in \Gamma } |\langle \pi (\gamma )^* g, \pi (x) g \rangle |^2 \; d\mu _{G/\Gamma } (x\Gamma ) \\\le & {} {{\,\mathrm{vol}\,}}(G/\Gamma ) B \Vert g \Vert _{\mathcal {H}_{\pi }}^2 < \infty . \end{aligned}$$

An irreducible $\pi$ with a non-zero $L^2$-integrable matrix coefficient is called a (projective) *discrete series*; see Section 2.3 for several basic properties. Since nilpotent and (unimodular) exponential Lie groups do not admit genuine representations that are square-integrable in the strict sense, the use of projective representations is particularly convenient; see Section 3.

In [2, 6], it has been shown that the existence of frames and Riesz sequences of the form $\pi (\Gamma ) g$ can be completely characterized in terms of properties of an associated $\sigma$-twisted convolution operator on $\ell ^2 (\Gamma )$, with $\sigma : G \times G \rightarrow \mathbb {T}$ being the 2-cocycle of the projective representation $\pi$. An element $\gamma \in \Gamma$ (and its conjugacy class $C_{\Gamma }(\gamma )$ in $\Gamma$) is called $\sigma$-*regular* in $\Gamma$ if $\gamma$ satisfies $\sigma (\gamma , \gamma ') = \sigma (\gamma ', \gamma )$ whenever $\gamma ' \in Z_{\Gamma }(\gamma )$, where $Z_{\Gamma }(\gamma )$ denotes the centralizer of $\gamma$ in $\Gamma$.

The following theorem contains the main results of [2, 6] for frames and Riesz sequences.

### Theorem 1.1

([2, 6]). Let $(\pi , \mathcal {H}_{\pi })$ be a discrete series $\sigma$-representation of *G* of formal degree $d_{\pi } > 0$. Let $\Gamma \le G$ be a lattice. For a unit vector $\eta \in \mathcal {H}_{\pi }$, define $\phi : \Gamma \rightarrow \mathbb {C}$ by

$$\begin{aligned} \phi (\gamma )= {\left\{ \begin{array}{ll} \dfrac{d_{\pi }}{|C_{\Gamma } (\gamma )|} \displaystyle \int _{G/Z_{\Gamma }(\gamma )} \overline{\sigma (\gamma , y)} \sigma (y, y^{-1} \gamma y)\\ \quad \cdot \; \langle \eta , \pi (y^{-1} \gamma y) \eta \rangle {\mathrm {d}}{(yZ_{\Gamma }(\gamma ))}, &{}{} \begin{array}{l} \text{ if }\, C_{\Gamma }(\gamma )\, \text{ is } \text{ finite } \\ \text{ and }\, \sigma \text{-regular; } \end{array} \\ 0, &{}{} \text{ otherwise }. \end{array}\right. } \end{aligned}$$

Let $C_{\phi }$ be the $\sigma$-twisted convolution operator on $\ell ^2 (\Gamma )$ defined by

1.3 $$\begin{aligned} (C_{\phi } c)(\gamma ') := \sum _{\gamma \in \Gamma } \phi (\gamma ) \sigma (\gamma , \gamma ^{-1} \gamma ') c(\gamma ^{-1} \gamma '), \quad \gamma ' \in \Gamma , \; c \in \ell ^2 (\Gamma ). \end{aligned}$$

Then the following assertions hold:

(i) There exists $g \in \mathcal {H}_{\pi }$ such that $\pi (\Gamma ) g$ is a frame if and only if $C_{\phi } \le I_{\ell ^2}$.
(ii) There exists $g \in \mathcal {H}_{\pi }$ such that $\pi (\Gamma ) g$ is a Riesz sequence if and only if $C_{\phi } \ge I_{\ell ^2}$.

The convolution operator $C_{\phi }$ defined in Theorem 1.1 determines the so-called *center-valued von Neumann dimension* or *coupling operator* of $\mathcal {H}_{\pi }$ as a module over the (twisted) group von Neumann algebra of $\Gamma$. Assertions (i) and (ii) in Theorem 1.1 are consequences of the underlying theory of von Neumann algebras. The paper [2] provides the statements of Theorem 1.1 for genuine representations and frames (cf. [2, Theorem 1]), and [6] provides an extension to possibly projective representations and Riesz sequences (cf. [6, Theorem 1.1]).

As a direct consequence of Theorem 1.1, one obtains the following necessary “density conditions”.

### Corollary 1.2

With the assumptions and notations as in Theorem 1.1,

(i) If there exists $g \in \mathcal {H}_{\pi }$ such that $\pi (\Gamma ) g$ is a frame, then ${{\,\mathrm{vol}\,}}(G/\Gamma ) d_{\pi } \le 1$.
(ii) If there exists $g \in \mathcal {H}_{\pi }$ such that $\pi (\Gamma ) g$ is a Riesz sequence, then ${{\,\mathrm{vol}\,}}(G/\Gamma ) d_{\pi } \ge 1$.

For a simple proof of Corollary 1.2 based on frame and representation theory, see [16].

The density conditions provided by Corollary 1.2 are generally not sharp, in the sense that they are not sufficient for the existence of frames and Riesz sequences of the form $\pi (\Gamma ) g$. For example, this might fail for discrete series of semi-simple Lie groups with a non-trivial center, cf. [2, Example 1]. However, for semi-simple Lie groups with a trivial center, the convolution kernel $\phi$ of the operator $C_{\phi }$ in Theorem 1.1 is simply given by

1.4 $$\begin{aligned} \phi = {{\,\mathrm{vol}\,}}(G/\Gamma ) d_{\pi } \cdot \delta _e . \end{aligned}$$

In general, if the identity (1.4) holds, then the density conditions provided by Corollary 1.2 are also sufficient for the existence of frames and Riesz sequences. In particular, this holds for lattices in which every non-trivial $\sigma$-regular conjugacy class has infinite cardinality; such pairs $(\Gamma , \sigma )$ are sometimes said to satisfy “Kleppner’s condition” [11].

It is the aim of this note to provide new criteria under which the convolution kernel $\phi$ in Theorem 1.1 takes the simple form (1.4). In particular, this will imply the optimality of the density conditions provided by Corollary 1.2.

In order to state the key result of this note, let *B*(*G*) be the set of all elements with pre-compact conjugacy classes in *G*. Then *B*(*G*) is a normal subgroup of *G* containing the center *Z*(*G*) and was studied for classes of locally compact groups in, e.g., [8, 13, 18, 21]. In particular, it was shown that exponential solvable Lie groups and reductive algebraic groups (with no simple factors) have the property $B(G) = Z(G)$, cf. Example 2.1 for references.

The following result provides criteria for a unimodular *G* with $B(G) = Z(G)$ under which the convolution operator $C_{\phi }$ of Theorem 1.1 is a scalar multiple of the identity operator.

### Theorem 1.3

Let *G* be such that $B(G) = Z(G)$ and let $\Gamma \le G$ be a lattice. Suppose that either *G* is locally connected or $\Gamma$ is co-compact. Suppose $(\pi , \mathcal {H}_{\pi })$ is a discrete series of formal degree $d_{\pi } > 0$ such that the projective kernel $P_{\pi } := \{ x \in G : \pi (x) \in \mathbb {C} \cdot I_{\mathcal {H}_{\pi }} \}$ is trivial. Then the twisted convolution operator $C_{\phi }$ on $\ell ^2 (\Gamma )$ defined in (1.3) is given by

1.5 $$\begin{aligned} C_{\phi } = {{\,\mathrm{vol}\,}}(G/\Gamma )d_{\pi } \cdot I_{\ell ^2}. \end{aligned}$$

Consequently, the following assertions hold:

(i) If ${{\,\mathrm{vol}\,}}(G/\Gamma ) d_{\pi } \le 1$, then there exists a frame $\pi (\Gamma ) g$ for $\mathcal {H}_{\pi }$.
(ii) If ${{\,\mathrm{vol}\,}}(G/\Gamma ) d_{\pi } \ge 1$, then there exists a Riesz sequence $\pi (\Gamma ) g$ in $\mathcal {H}_{\pi }$.

Theorem 1.3 is applicable to all cases in which the necessary density conditions of Corollary 1.2 are known to be sharp, namely for Abelian groups [6, Corollary 4.6], linear algebraic semi-simple groups [2, Theorem 2], and square-integrable representations modulo the center of nilpotent Lie groups [2, Theorem 3]. In addition, it is applicable to exponential Lie groups and reductive algebraic groups and it allows to treat representations that are only square-integrable modulo their projective kernel since any such representation is naturally treated as a projective discrete series of the quotient (cf. Section 3).

The assumption in Theorem 1.3 that the projective kernel $P_{\pi }$ is trivial is essential for its validity. For example, both conclusions (i) and (ii) fail for a holomorphic discrete series $\pi$ of $\mathrm {SL}(2, \mathbb {R})$ (cf. [2, Example 2]), where $\{-I, I \} \subseteq P_{\pi }$, but Theorem 1.3 is applicable to the (projective) discrete series of $\mathrm {PSL}(2, \mathbb {R}) = \mathrm {SL}(2, \mathbb {R}) / \{ -I, I \}$. On the other hand, for the existence of Riesz sequences $\pi (\Gamma ) g$ in general, it is necessary that $\pi |_{\Gamma }$ acts projectively faithful.

A particular motivation for obtaining Theorem 1.3 was to investigate the optimality of the density conditions in Corollary 1.2 for the existence of frames and Riesz sequences for general exponential Lie groups, i.e., Lie groups for which the exponential map is a diffeomorphism. For a description of the projective discrete series of an exponential Lie group in terms of the Kirillov correspondence, see [12, 20]; in particular, cf. [20, Proposition 4].

### Theorem 1.4

Let *G* be an exponential solvable Lie group and let $\Gamma \le G$ be a lattice. Let $(\pi , \mathcal {H}_{\pi })$ be a projective discrete series of *G* of formal degree $d_{\pi } > 0$. Then the conclusions of Theorem 1.3 hold.

Theorem 1.4 covers, in particular, projective representations obtained from genuine representations that are square-integrable modulo the center (see Remark 3.2). The existence of frames for such representations of nilpotent Lie groups was shown in [2] (cf. [2, Theorem 3] and [2, Corollary 4]). A statement on Riesz sequences does not seem to follow easily from [2, Theorem 3]. On the other hand, the existence of Riesz sequences follows transparently from Theorem 1.4, which makes it relevant even for the special case of nilpotent groups.

A non-nilpotent example to which Theorem 1.4 is applicable is given in Section 4.

## Restrictions of discrete series to lattices

Throughout, unless stated otherwise, *G* denotes a second-countable unimodular locally compact group. A fixed Haar measure on *G* will be denoted by $\mu _G$. If $H \le G$ is a closed subgroup with Haar measure $\mu _H$, then there exists a unique *G*-invariant Radon measure $\mu _{G/H}$ on the space *G*/*H* of left cosets of *H* such that Weil’s formula holds:

2.1 $$\begin{aligned} \int \limits _{G} f(x) {\mathrm{d}}{\mu _G(x)} = \int \limits _{G/H} \int \limits _{H} f(xy) {\mathrm{d}}{\mu _H(y)} {\mathrm{d}}{\mu _{G/H}(xH)} , \quad f \in L^1(G). \end{aligned}$$

The measure $\mu _{G/H}$ will always be assumed to be normalized such that (2.1) holds. If *H* is discrete, then $\mu _H$ will be assumed to be the counting measure.

### Bounded conjugacy classes

For a subset $S \subseteq G$, the centralizer of *S* in *G* is denoted by $Z_G (S) = \{ x \in G: xs = sx, \; \forall s \in S \}$. In particular, we write $Z_G(x) = Z_G(\{ x \})$ for $x \in G$, and $Z(G) = Z_G (G)$. For $x \in G$, its conjugacy class is $C_G(x) = \{ y x y^{-1} : y \in G \}$. The map $y Z_G(x) \mapsto y x y^{-1}$ is a continuous bijection from $G/Z_G(x)$ onto $C_G(x)$.

The conjugacy class $C_G (x)$ of $x \in G$ is called *bounded* if its closure is compact. The set of all elements $x \in G$ for which $C_G (x)$ is bounded will be denoted by *B*(*G*). The set *B*(*G*) is a normal subgroup in *G* containing the center *Z*(*G*).

An automorphism $\alpha \in {{\,\mathrm{Aut}\,}}(G)$ is said to be of *bounded displacement* if $\{ x^{-1} \alpha (x) : x \in G \}$ is pre-compact. It is readily verified that an inner automorphism $\alpha _y : G \rightarrow G, \; x \mapsto y^{-1} x y$, is of bounded displacement if and only if $y \in B(G)$. If *G* admits no non-trivial automorphisms of bounded displacement, then $B(G) = Z(G)$.

Locally compact groups *G* for which $B(G) = Z(G)$ will play a key role in this note. The following example lists (classes of) groups for which this condition is satisfied.

#### Example 2.1

The condition $B(G) = Z(G)$ holds in each of the following cases:

- Abelian groups.
- Connected, simply connected nilpotent Lie groups (cf. [21, Theorem 1]).
- Exponential solvable Lie groups *G*, i.e., the exponential map $\exp : \mathfrak {g} \rightarrow G$ is a diffeomorphism (cf. [8, Theorem 9.4] or [13, Corollary 1.3]).
- Connected, simply connected complex analytic Lie groups (cf. [8, Theorem 9.4]).
- Connected semi-simple Lie groups with no compact factors (cf. [8, Theorem 9.1]).
- Connected reductive linear algebraic groups with no simple factors (cf. [18, Theorem 2.4]).

### Lattices

A discrete subgroup $\Gamma \le G$ is said to be a *lattice* if the unique invariant Radon measure on $G/\Gamma$ provided by (2.1) is finite. A lattice $\Gamma$ is called *uniform* if $G/\Gamma$ is compact. For classes of amenable groups, including connected solvable Lie groups, any lattice is automatically uniform, see [1, 14].

#### Lemma 2.2

Let *G* be such that $B(G) = Z(G)$ and let $\Gamma \le G$ be a lattice. Suppose that either *G* is locally connected or that $\Gamma \le G$ is uniform. Then the following assertions hold:

(i) For every $\gamma \in \Gamma$, the conjugacy class $C_{\Gamma } (\gamma )$ in $\Gamma$ is either trivial or infinite.
(ii) The centralizer $Z_G(\Gamma )$ of $\Gamma$ in *G* equals the center *Z*(*G*) of *G*.

#### Proof

(i) Let $\gamma \in \Gamma$ be such that $C_{\Gamma } (\gamma )$ is finite. Then $\Gamma /Z_{\Gamma }(\gamma )$ is also finite.

First, suppose that *G* is locally connected. Let $\mu _{G/Z_{\Gamma } (\gamma )}$, $\mu _{G/\Gamma }$, and $\mu _{\Gamma / Z_{\Gamma } (\gamma )}$ be the invariant Radon measures on the coset spaces $G/Z_{\Gamma }(\gamma )$, $G/\Gamma$, and $\Gamma /Z_{\Gamma }(\gamma )$, respectively. Since $\mu _{G/\Gamma }$ and $\mu _{\Gamma / Z_{\Gamma } (\gamma )}$ are finite, it follows that also $\mu _{G/Z_{\Gamma } (\gamma )}$ is finite, see, e.g., [15, Lemma 1.6]. Hence, since $Z_{\Gamma }(\gamma ) \subseteq Z_G(\gamma )$, the continuous map $G/Z_{\Gamma }(\gamma ) \rightarrow G/Z_G(\gamma )$ yields a finite *G*-invariant measure on $G/Z_G(\gamma )$. An application of [19, Theorem] yields that $G/Z_G(\gamma )$ is compact. Hence, $C_G(\gamma ) \cong G/Z_G(\gamma )$ is compact, so that $\gamma \in B(G) = Z(G)$.

Secondly, if $\Gamma$ is a uniform lattice, then there exists a compact set $\Omega \subseteq G$ such that $G = \Omega \cdot \Gamma$. The conjugacy class $C_G (\gamma )$ is therefore given by

$$\begin{aligned} C_G (\gamma ) = \{ x \gamma x^{-1} : x \in G \} \subseteq \Omega \cdot C_{\Gamma } (\gamma ) \cdot \Omega ^{-1}, \end{aligned}$$

whence pre-compact in *G*. Thus $\gamma \in B(G) = Z(G)$, and hence $C_{\Gamma } (\gamma ) = \{\gamma \}$.

(ii) Let $x \in Z_G(\Gamma )$, so that $\Gamma \subseteq Z_G(x)$. Suppose first that *G* is locally connected. The finite *G*-invariant measure on $G/\Gamma$ can be pushed forward to a finite *G*-invariant measure on $G/Z_G(x)$, which implies that $G/Z_G(x)$ is compact by [19, Theorem]. Hence, $C_G(x) \cong G/Z_G(x)$ is compact, and thus $x \in B(G) = Z(G)$.

Lastly, if $\Gamma$ is a uniform lattice, then the continuous surjective map $G / \Gamma \rightarrow G/Z_G(x)$ yields that $G/Z_G(x)$ is compact. Therefore, the continuous bijection $G/Z_G(x) \rightarrow C_G(x)$ yields that also $C_G (x)$ is compact, and thus $x \in B(G) = Z(G)$. $\square$

Lemma 2.2 applies, in particular, to arbitrary lattices in Lie groups. For this setting, there are alternative proofs of the used [19, Theorem], see [9, Theorem 1] and [7, Theorem 2]. It is not known whether Lemma 2.2 holds for non-uniform lattices in general unimodular groups.

### Projective discrete series

A *projective unitary representation* $(\pi , \mathcal {H}_{\pi })$ of *G* on a separable Hilbert space $\mathcal {H}_{\pi }$ is a strongly measurable map $\pi : G \rightarrow \mathcal {U}(\mathcal {H}_{\pi })$ satisfying

$$\begin{aligned} \pi (x)\pi (y) = \sigma (x,y)\pi (xy), \quad x,y \in G, \end{aligned}$$

for some function $\sigma : G \times G \rightarrow \mathbb {T}$. The function $\sigma$ necessarily forms a 2-*cocycle* on *G*, that is, it is a Borel function satisfying the identities

$$\begin{aligned} \sigma (e, e) = 1 \quad \text {and} \quad \sigma (x,y)\sigma (xy,z)&= \sigma (x,yz)\sigma (y,z) \quad \text {for all}\, x,y,z \in G. \end{aligned}$$

A projective unitary representation with 2-cocycle $\sigma$ will simply be referred to as a $\sigma$-representation. For $\sigma \equiv 1$, it will simply be said that $\pi$ is a *representation*.

A $\sigma$-representation $(\pi , \mathcal {H}_{\pi })$ is *irreducible* if the only closed $\pi (G)$-invariant subspaces are $\{0\}$ and $\mathcal {H}_{\pi }$. It is called *square-integrable* if there exist nonzero $f,g \in \mathcal {H}_{\pi }$ such that

$$\begin{aligned} \int \limits _{G} | \langle f, \pi (x) g \rangle |^2 \; d \mu _G (x) < \infty . \end{aligned}$$

An irreducible, square-integrable $\sigma$-representation is called a *discrete series* $\sigma$*-representation*, or a *projective discrete series* if the associated cocycle is irrelevant.

The significance of a discrete series $\pi$ is the existence of a unique $d_{\pi } > 0$, called its *formal degree*, such that the orthogonality relations

$$\begin{aligned} \int \limits _{G} \langle f, \pi (x) g \rangle \overline{ \langle f', \pi (x) g' \rangle } \; d \mu _G (x) = d_{\pi }^{-1} \langle f, f' \rangle \overline{ \langle g, g' \rangle } \end{aligned}$$

hold for all $f,f',g,g' \in \mathcal {H}_{\pi }$.

### The projective kernel

The *projective kernel* of a $\sigma$-representation $\pi$ is defined by

2.2 $$\begin{aligned} P_{\pi } := \{ x \in G : \pi (x) \in \mathbb {T}\cdot I_{\mathcal {H}_{\pi }} \}. \end{aligned}$$

The $\sigma$-representation $\pi$ is *projectively faithful* if $P_{\pi } = \{e\}$.

Throughout, $\chi _\pi : P_{\pi } \rightarrow \mathbb {T}$ denotes the measurable function satisfying $\pi (x) = \chi _\pi (x)I_{\mathcal {H}_{\pi }}$ for all $x \in P_{\pi }$. Then, for $f,g \in \mathcal {H}_{\pi }$, $x \in G$, and $y \in P_{\pi }$,

2.3 $$\begin{aligned} |\langle f, \pi (xy) g \rangle | = |\langle f, \overline{\sigma (x,y)} \pi (x) \chi _\pi (y) g \rangle | = | \langle f, \pi (x) g \rangle |, \end{aligned}$$

so that $xP_{\pi }\mapsto |\langle f, \pi (x) g \rangle |$ is a well-defined function on the coset space $G/P_{\pi }$.

#### Lemma 2.3

If $\pi$ is a $\sigma$-representation of *G*, then $P_{\pi }$ is a closed normal subgroup. If, in addition, $\pi$ is square-integrable, then $P_{\pi }$ is compact.

#### Proof

Let $\mathcal {P}(\mathcal {H}_{\pi }) := \mathcal {U}(\mathcal {H}_{\pi }) / \mathbb {T} \cdot I_{\mathcal {H}_{\pi }}$ be the projective unitary group of $\mathcal {H}_{\pi }$, equipped with the quotient topology relative to the strong operator topology on $\mathcal {U}(\mathcal {H}_{\pi })$. Let $p : \mathcal {U}(\mathcal {H}_{\pi }) \rightarrow \mathcal {P}(\mathcal {H}_{\pi })$ be the canonical projection. By [22, Theorem 7.5], the map $\pi ' := p \circ \pi : G \rightarrow \mathcal {P}(\mathcal {H}_{\pi })$ is a continuous homomorphism, and hence $P_{\pi } = \ker (\pi ')$ is a closed normal subgroup.

Suppose $\pi$ is square-integrable. Letting $f,g \in \mathcal {H}_{\pi }\setminus \{0\}$, we apply (2.1) and (2.3) to obtain

$$\begin{aligned} \infty > \int \limits _{G} | \langle f, \pi (x) g \rangle |^2 {\mathrm{d}}{\mu _G(x)} = \int \limits _{P_{\pi }} {\mathrm{d}}{\mu _{P_{\pi }}(y)} \int \limits _{G/P_{\pi }} |\langle f, \pi (x) g \rangle |^2 {\mathrm{d}}{\mu _{G/P_{\pi }}(xP_{\pi })}, \end{aligned}$$

and thus the Haar measure of $P_{\pi }$ is finite, so that $P_{\pi }$ must be compact. $\square$

Following [11], $x \in G$ is called $\sigma$-*regular* in *G* if $\sigma (x,y) = \sigma (y,x)$ for all $y \in Z_G (x)$.

#### Lemma 2.4

Let *G* be such that $B(G) = Z(G)$ and let $(\pi , \mathcal {H}_{\pi })$ be a discrete series $\sigma$-representation of *G*. Then the projective kernel coincides with the $\sigma$-regular elements of the center of *G*. In particular, the following are equivalent:

(i) $\pi$ is projectively faithful.
(ii) The only $\sigma$-regular element of *G* with precompact conjugacy class is the identity.

#### Proof

If an element $x \in Z(G)$ is $\sigma$-regular, then

$$\begin{aligned} \pi (x)\pi (y) = \sigma (x,y) \pi (xy) = \sigma (y,x) \pi (yx) = \pi (y)\pi (x) \end{aligned}$$

for all $y \in G$. Thus $\pi (x) \in \pi (G)' = \mathbb {T}\cdot I_{\mathcal {H}_{\pi }}$ by irreducibility, so that $x \in P_{\pi }$. Conversely, if $x \in P_{\pi }$, then $C_G(x) \subseteq P_{\pi }$ since $P_{\pi }$ is a normal subgroup of *G*. Since $P_{\pi }$ is compact by Lemma 2.3, it follows that $C_G(x)$ is pre-compact, hence $x \in B(G) = Z(G)$. Therefore, for any $y \in G$,

$$\begin{aligned} \chi _\pi (x)\pi (y)&= \pi (x)\pi (y) = \sigma (x,y)\pi (xy) = \sigma (x,y) \pi (yx) \\&= \sigma (x,y)\overline{\sigma (y,x)} \pi (y)\pi (x) = \sigma (x,y)\overline{\sigma (y,x)} \chi _\pi (x) \pi (y) . \end{aligned}$$

Hence $\sigma (x,y) = \sigma (y,x)$ for all $y \in G$, so *x* is $\sigma$-regular.

Since $B(G) = Z(G)$, it follows that the projective kernel coincides with $\sigma$-regular elements with pre-compact conjugacy classes. In particular, $P_{\pi } = \{ e \}$ if and only if the only $\sigma$-regular element with pre-compact conjugacy class is the identity. $\square$

A combination of the previous lemmata allows a proof of Theorem 1.3:

#### Proof of Theorem 1.3

Suppose $\gamma \in \Gamma$ is $\sigma$-regular in $\Gamma$ and $C_{\Gamma } (\gamma )$ is finite. For showing (1.5), we have to show that $\phi (\gamma ) = {{\,\mathrm{vol}\,}}(G/\Gamma )d_{\pi } \delta _{\gamma ,e}$. By Lemma 2.2(i), it follows that $C_{\Gamma } (\gamma )$ is trivial, so that $\gamma$ is in the center $Z(\Gamma )$ of $\Gamma$. In particular, this implies that $Z_{\Gamma } (\gamma ) = \Gamma$. In addition, Lemma 2.2(ii) yields that $\gamma \in Z(G)$. Hence,

$$\begin{aligned} \phi (\gamma )&= d_{\pi } \int \limits _{G/\Gamma } \overline{\sigma (\gamma , y)} \sigma (y, y^{-1} \gamma y) \langle \eta , \pi (y^{-1} \gamma y) \eta \rangle \; d\mu _{G/\Gamma } {(y\Gamma )} \\&= d_{\pi } \langle \eta , \pi (\gamma ) \eta \rangle \int \limits _{G/\Gamma } \overline{\sigma (\gamma ,y)} \sigma (y,\gamma ) \; d \mu _{G/\Gamma } {(y\Gamma )}. \end{aligned}$$

The function $\omega _{\gamma } :G \rightarrow \mathbb {T}$ given by $\omega _{\gamma } (y) = \overline{\sigma (\gamma ,y)} \sigma (y,\gamma )$ is a homomorphism since

$$\begin{aligned}&\omega _{\gamma }(yy') = \overline{\sigma (\gamma ,yy')} \sigma (yy',\gamma ) = \overline{\sigma (\gamma ,yy') \sigma (y,y')} \sigma (y,y') \sigma (yy',\gamma ) \\&= \overline{\sigma (\gamma ,y)\sigma (\gamma y,y')} \sigma (y,y'\gamma ) \sigma (y',\gamma ) \\&= \overline{ \sigma (\gamma ,y)\sigma (y \gamma ,y') \sigma (\gamma ,y')} \sigma (y,\gamma y') \sigma (\gamma ,y') \sigma (y',\gamma ) \\&= \overline{\sigma (\gamma ,y)\sigma (y\gamma ,y') \sigma (\gamma ,y')} \sigma (y,\gamma )\sigma (y\gamma ,y') \sigma (y',\gamma ) \\&= \overline{\sigma (\gamma ,y) } \sigma (y,\gamma ) \overline{ \sigma (\gamma ,y')} \sigma (y',\gamma ) \\&= \omega _{\gamma } (y)\omega _{\gamma } (y'). \end{aligned}$$

The *G*-invariance of the measure on $G/\Gamma$ gives that

$$\begin{aligned}&\int \limits _{G/\Gamma } \omega _{\gamma } (y) {\mathrm{d}}{\mu _{G/\Gamma }(y\Gamma )} = \int \limits _{G/\Gamma } \omega _{\gamma } (y'y) {\mathrm{d}}{\mu _{G/\Gamma }(y\Gamma )}\\&\quad = \omega _{\gamma } (y') \int \limits _{G/\Gamma } \omega _{\gamma } (y) {\mathrm{d}}{\mu _{G/\Gamma }(y\Gamma )} , \quad y' \in G, \end{aligned}$$

which means that

$$\begin{aligned} \int \limits _{G/\Gamma } \omega _{\gamma } (y\Gamma ) \; d{\mu _{G/\Gamma }(y\Gamma )} = {\left\{ \begin{array}{ll} {{\,\mathrm{vol}\,}}(G/\Gamma ), &{} \text {if}\, \omega _{\gamma } \equiv 1, \\ 0, &{} \text {otherwise.} \end{array}\right. } \end{aligned}$$

Note that $\omega _{\gamma } \equiv 1$ if and only if $\sigma (\gamma ,y) = \sigma (y,\gamma )$ for all $y \in G$, i.e., if and only if $\gamma$ is $\sigma$-regular in *G*. Since $\gamma \in Z(G)$, $\gamma$ is $\sigma$-regular in *G* if and only if $\gamma = e$ by Lemma 2.4 and the assumption that $\pi$ is projectively faithful. Hence,

$$\begin{aligned} \phi (\gamma ) = {{\,\mathrm{vol}\,}}(G/\Gamma ) d_{\pi } \cdot \delta _{\gamma ,e} , \end{aligned}$$

as required. $$\square $$

#### Proof of Theorem 1.4

Since *G* is exponential, we have $Z(G) = B(G)$ by [8, Theorem 9.4]. By Lemma 2.3, the projective kernel $P_{\pi }$ of a discrete series $\pi$ must be compact, hence trivial, since *G* does not contain nontrivial compact subgroups, see, e.g., [10, Theorem 14.3.12]. The conclusion follows therefore directly from Theorem 1.3. $\square$

## Discrete series modulo the projective kernel

This section considers projective representations obtained from genuine representations that are square-integrable modulo their projective kernel. Such projective representations are projectively faithful, and they form an important class to which Theorem 1.3 applies.

Let $(\rho ,\mathcal {H}_{\rho })$ be an irreducible representation of a second countable unimodular group *H*. It is called a *relative discrete series* (modulo $P_{\rho }$) if there exist non-zero $f, g \in \mathcal {H}_{\rho }$ such that

$$\begin{aligned} \int \limits _{H/P_{\rho }}|\langle f, \rho (\dot{x}) g \rangle |^2 \; d\mu _{H/P_{\rho }} (\dot{x}) < \infty , \end{aligned}$$

where $\dot{x} = xP_{\rho }$ and $\mu _{H/P_{\rho }}$ denotes the Haar measure on $H/P_{\rho }$.

A relatively discrete series $(\rho , \mathcal {H}_{\rho })$ of *H* can be treated as a (projective) discrete series of $G := H / P_{\rho }$. For this, choose a Borel section $s :H/P_{\rho }\rightarrow H$ of the canonical quotient map, and set $\pi := \rho \circ s$. Then a direct calculation shows that

$$\begin{aligned} \pi (\dot{x})\pi (\dot{y}) = \sigma (\dot{x},\dot{y})\pi (\dot{x} \dot{y}) , \quad \dot{x}, \dot{y} \in G = H/P_{\rho }, \end{aligned}$$

where the 2-cocycle $\sigma$ is given by

$$\begin{aligned} \sigma (\dot{x},\dot{y}) = \chi _{\rho } ( s(\dot{x})s(\dot{y})s(\dot{x} \dot{y})^{-1}), \quad \dot{x}, \dot{y} \in G = H/P_{\rho }. \end{aligned}$$

A different choice of the section *s* yields a 2-cocycle cohomologous to $\sigma$ and a representation unitarily equivalent to $\pi$.

The following proposition is a special case of Theorem 1.3.

### Proposition 3.1

Let $(\rho , \mathcal {H}_{\rho })$ be a relative discrete series (modulo $P_{\rho }$) of a unimodular group *H*. Suppose that $G = H / P_{\rho }$ is unimodular and denote by $\pi$ a $\sigma$-representation of *G* associated to $\rho$. Let $\Gamma \le G$ be a lattice. If $B(G) = Z(G)$ and either *G* is locally connected or $\Gamma$ is uniform, then $C_{\phi } = {{\,\mathrm{vol}\,}}(G/\Gamma )d_{\pi } \cdot I_{\ell ^2} .$

### Proof

Denote by $\pi$ a $\sigma$-representation of $G = H /P_{\rho }$ obtained from $\rho$ via a Borel section *s*. If $xP_{\rho }\in P_{\pi }\le H/P_{\rho }$, then $\rho (s(xP_{\rho })) = \pi (xP_{\rho }) \in \mathbb {T}\cdot I_{\mathcal {H}_{\pi }}$, so that $s(xP_{\rho }) \in P_{\rho }$. This implies that $xP_{\rho }= s(xP_{\rho }) P_{\rho }= P_{\rho }$, and thus $P_{\pi }= \{ e P_{\rho }\}$. Therefore, it follows from Theorem 1.3 that $C_{\phi } = {{\,\mathrm{vol}\,}}(G/\Gamma ) d_{\pi } \cdot I_{\ell ^2}$.

$$\square $$

### Remark 3.2

The projective kernel of an irreducible representation $(\rho , \mathcal {H}_{\rho })$ of an exponential Lie group *H* is connected by [3, Theorem 2.1], and thus $G= H/P_{\rho }$ is again exponential and $B(G) = Z(G)$ by [8, Theorem 9.4]. In the particular case that $\rho$ is square-integrable modulo the center *Z*(*H*), then $P_{\rho } = Z(H)$ by [3, Theorem 2.1] combined with [5, Theorem 5.3.4] or [17, Section 4.1]. Especially, if *H* is unimodular, then $G = H/Z(H)$ is unimodular.

## A non-nilpotent exponential ${ {\hbox {SI/Z}}}$ group with a lattice.

This section provides an example of a non-nilpotent exponential Lie group to which Theorem 1.4 is applicable, showing that it is not vacuous in the non-nilpotent case.

### Completely solvable group

Let $\mathfrak {g} = {{\,\mathrm{span}\,}}_{\mathbb {R}} \{X_1, ..., X_5\}$ with non-zero Lie brackets

$$\begin{aligned}{}[X_2, X_3]=X_1, \; [X_2, X_5] = X_2, \; [X_3, X_5 ] = - X_3, \; [X_4, X_5] = X_1. \end{aligned}$$

Then $\mathfrak {g}$ is completely solvable, i.e., it admits a sequence of ideals

$$\begin{aligned} \{ 0 \} = \mathfrak {g}_0 \subset \mathfrak {g}_1 \subset \cdots \subset \mathfrak {g}_{4} \subset \mathfrak {g}_5 = \mathfrak {g}, \quad \text {with} \quad \dim (\mathfrak {g}_j) = j. \end{aligned}$$

In particular, this shows that $\mathfrak {g}$ is an exponential solvable Lie algebra. Its nilradical is given by $\mathfrak {n} = {{\,\mathrm{span}\,}}_{\mathbb {R}} \{X_1, X_2, X_3 \} \oplus \mathbb {R} X_4$, so that $\mathfrak {g}$ is non-nilpotent. The center of $\mathfrak {g}$ is $\mathfrak {z} (\mathfrak {g}) = \mathbb {R} X_1$.

Let *G* be the connected, simply connected Lie group with Lie algebra $\mathfrak {g}$. Denote by *N* and *T* the connected Lie subgroups with Lie algebras $\mathfrak {n}$ and $\mathbb {R} X_5$, respectively. Then *G* is a semi-direct product $G = N T$ with group multiplication

$$\begin{aligned} \begin{pmatrix} x \\ y \\ z \\ w \\ t \end{pmatrix} \cdot \begin{pmatrix} x' \\ y' \\ z' \\ w' \\ t' \end{pmatrix} = \begin{pmatrix} x +x' - t w' - \frac{1}{2}(e^t z y' - e^{-t} y z') \\ y + e^{-t} y' \\ z + e^t z' \\ w + w' \\ t + t' \end{pmatrix}. \end{aligned}$$

The center of *G* is given by $Z(G) = \{ (x,0,0,0,0) : x \in \mathbb {R} \}$.

### Lattice

A lattice in *G* can be given as follows, cf. [4, p. 237]: Let $n \in \mathbb {N}$, $n > 2$, and let $t_0 > 0$ be such that $(e^{t_0})^2 - ne^{t_0} + 1 = 0$. Let $v_1 = (1,1)$ and $v_2 = (e^{-t_0},e^{t_0})$ and $x_0 = (e^{-t_0} - e^{t_0})/2$. Then the set

$$\begin{aligned} \Gamma = \mathbb {Z}\begin{pmatrix} x_0 \\ 0 \\ 0 \\ 0 \\ 0 \end{pmatrix} + \mathbb {Z}\begin{pmatrix} 0 \\ v_1 \\ 0 \\ 0 \end{pmatrix} + \mathbb {Z}\begin{pmatrix} 0 \\ v_2 \\ 0 \\ 0 \end{pmatrix} + \mathbb {Z}\begin{pmatrix} 0 \\ 0 \\ 0 \\ x_0/t_0 \\ 0 \end{pmatrix} + \mathbb {Z}\begin{pmatrix} 0 \\ 0 \\ 0 \\ 0 \\ t_0 \end{pmatrix} \end{aligned}$$

forms a lattice in *G*.

The projection of this lattice via the quotient map to *G*/*Z*(*G*) yields a lattice in *G*/*Z*(*G*).

### Relative discrete series

For showing that *G* admits relative discrete series representations, it suffices (cf. [17, §4.1]) to show that there exists a functional $\ell \in \mathfrak {g}^*$ such that

$$\begin{aligned} \mathfrak {g} (\ell ) := \{X \in \mathfrak {g} : \ell ([Y,X]) = 0, \; \forall \; Y \in \mathfrak {g} \} = \mathfrak {z}(\mathfrak {g}). \end{aligned}$$

Denoting by $(\xi _1, ..., \xi _5)$ a dual basis of $\{X_1, ..., X_5\}$ in $\mathfrak {g}^*$, it is readily verified that $\mathfrak {g}(\xi _1) = \mathfrak {z}(\mathfrak {g})$.

For $\xi := \xi _1 \in \mathfrak {g}^*$, consider the associated polarization $\mathfrak {p} = {{\,\mathrm{span}\,}}_{\mathbb {R}} \{X_1, X_2, X_4 \}$. Then

$$\begin{aligned} \chi _{\xi } (\exp (X)) = e^{2\pi i \xi (X)}, \quad X \in \mathfrak {p}, \end{aligned}$$

defines a unitary character of $P := \exp (\mathfrak {p}) \le G$. The Kirillov-Bernat correspondence yields that the induced representation $\rho _{\xi } = {{\,\mathrm{ind}\,}}_P^G (\chi _{\xi })$ forms an irreducible representation of *G* that is square-integrable modulo *Z*(*G*).
